# Pulmonary Toxicity After Total Body Irradiation – Critical Review of the Literature and Recommendations for Toxicity Reporting

**DOI:** 10.3389/fonc.2021.708906

**Published:** 2021-08-26

**Authors:** Jennifer Vogel, Susanta Hui, Chia-Ho Hua, Kathryn Dusenbery, Premavarthy Rassiah, John Kalapurakal, Louis Constine, Natia Esiashvili

**Affiliations:** ^1^Department of Radiation Oncology, Bon Secours Merch Health St. Francis Cancer Center, Greenville, SC, United States; ^2^Department of Radiation Oncology, City of Hope National Medical Center, Duarte, CA, United States; ^3^Department of Radiation Oncology, St Jude Children’s Research Hospital, Memphis, TN, United States; ^4^Department of Radiation Oncology, University of Minnesota, Minneapolis, MN, United States; ^5^Department of Radiation Oncology, University of Utah Huntsman Cancer Hospital, Salt Lake City, UT, United States; ^6^Department of Radiation Oncology, Northwestern University School of Medicine, Chicago, IL, United States; ^7^Department of Radiation Oncology, University of Rochester Medical Center, Rochester, NY, United States; ^8^Department of Radiation Oncology, Emory School of Medicine, Atlanta, GA, United States

**Keywords:** radiation pneumonitis, pulmonary toxicity, allogeneic stem cell transplantation, total body irradiation, total body irradiation complications

## Abstract

**Introduction:**

Total body irradiation is an effective conditioning regimen for allogeneic stem cell transplantation in pediatric and adult patients with high risk or relapsed/refractory leukemia. The most common adverse effect is pulmonary toxicity including idiopathic pneumonia syndrome (IPS). As centers adopt more advanced treatment planning techniques for TBI, total marrow irradiation (TMI), or total marrow and lymphoid irradiation (TMLI) there is a greater need to understand treatment-related risks for IPS for patients treated with conventional TBI. However, definitions of IPS as well as risk factors for IPS remain poorly characterized. In this study, we perform a critical review to further evaluate the literature describing pulmonary outcomes after TBI.

**Materials and Methods:**

A search of publications from 1960-2020 was undertaken in PubMed, Embase, and Cochrane Library. Search terms included “total body irradiation”, “whole body radiation”, “radiation pneumonias”, “interstitial pneumonia”, and “bone marrow transplantation”. Demographic and treatment-related data was abstracted and evidence quality supporting risk factors for pulmonary toxicity was evaluated.

**Results:**

Of an initial 119,686 publications, 118 met inclusion criteria. Forty-six (39%) studies included a definition for pulmonary toxicity. A grading scale was provided in 20 studies (17%). In 42% of studies the lungs were shielded to a set mean dose of 800cGy. Fourteen (12%) reported toxicity outcomes by patient age. Reported pulmonary toxicity ranged from 0-71% of patients treated with TBI, and IPS ranged from 1-60%. The most common risk factors for IPS were receipt of a TBI containing regimen, increasing dose rate, and lack of pulmonary shielding. Four studies found an increasing risk of pulmonary toxicity with increasing age.

**Conclusions:**

Definitions of IPS as well as demographic and treatment-related risk factors remain poorly characterized in the literature. We recommend routine adoption of the diagnostic workup and the definition of IPS proposed by the American Thoracic Society. Additional study is required to determine differences in clinical and treatment-related risk between pediatric and adult patients. Further study using 3D treatment planning is warranted to enhance dosimetric precision and correlation of dose volume histograms with toxicities.

## Introduction

Acute leukemia is the most common cancer in children and adolescents, and exhibits a bimodal distribution with an initial peak among infants and exponential rise in adulthood (1, 2). Between 2001-2007, 29,682 individuals were diagnosed with acute leukemia, with an incidence ratio of 57.2 per 100,000 person years (2). Overall, acute myeloid leukemia (AML) accounted for 65.7% of cases, acute lymphoblastic leukemia or lymphoma (ALL/L) 31.0% of cases, and acute leukemia of ambiguous lineage 3.4% of cases (2).

Allogeneic stem cell transplant is used in a subset of patients with high risk or relapsed/refractory disease. In pediatric patients with ALL, myeloablative regimens containing total body irradiation (TBI) have demonstrated improvement in event free survival from 29-35% without TBI as compared to 50-58% with and remain the standard of care (3–5). In the adult setting, myeloablative regimens have demonstrated improvements in recurrence free survival at the cost of increased transplant related mortality compared to reduced intensity conditioning regimens (6, 7). The use of reduced-intensity conditioning regimens has therefore increased over the last decade, particularly in patients 50 years of age and older (8).

Transplant-related morbidity and mortality following a myeloablative transplant is significant. In particular, pulmonary toxicity and mortality has been reported in up to 60% of patients (9). Historically, approximately half of all pneumonias following stem cell transplant were secondary to infection, but use of prophylaxis has resulted in a relatively greater risk from noninfectious etiologies (10). In 1993 the National Institutes of Health (NIH) defined idiopathic pneumonia syndrome (IPS) as widespread alveolar injury without evidence of active lower tract infection or cardiogenic cause after transplant (11). Updated definitions now include newly described pathogens as determined on bronchoalveolar lavage (BAL) or lung biopsy (**Table 1**) (10).

**Table 1:** Definitions of pulmonary toxicity.

National Institutes of Health, 1993 (1)	I. Evidence of widespread alveolar injury. Criteria include: a. Multilobar infiltrates on routine chest radiographics or CT scans. b. Symptoms and signs of pneumonia, e.g., cough, dyspnea, rales. c. Evidence of abnormal pulmonary physiology. i. Increased alveolar to arterial oxygen gradient ii. New or increased restrictive pulmonary function test abnormality II. Absence of active lower respiratory tract infection. Appropriate evaluation includes: a. BAL negative for significant bacterial pathogens and/or lack of improvement with broad-spectrum antibiotics. b. BAL negative for pathogenic nonbacterial microorganisms. i. Routine bacterial viral and fungal cultures. ii. Shell-vial CMV culture iii. Cytology for CMV inclusions, fungi, and PCP iv. Detection methods for RSV, para-influenza virus, and other organisms (e.g., fluorescent antibiotics or culture) c. Transbronchial biopsy if condition of the patient permits. d. Ideally, a second confirmatory negative test for infection is done. This is usually performed 2 to 14 days after the initial negative BAL and may consist of a second BAL or open lung biopsy.
American Thoracic Society (2)	I. Evidence of widespread alveolar injury. a. Multilobar infiltrates on routine chest radiographics or CT scans. b. Symptoms and signs of pneumonia, e.g., cough, dyspnea, rales. c. Evidence of abnormal pulmonary physiology. i. Increased alveolar to arterial oxygen difference ii. New or increased restrictive pulmonary function test abnormality II. Absence of active lower respiratory tract infection. Appropriate evaluation includes: a. BAL negative for significant bacterial pathogens including acid-fast bacilli, *Nocardia*, and *Legionella* species b. BAL negative for pathogenic nonbacterial microorganisms. i. Routine bacterial viral and fungal cultures. ii. Shell-vial for CMV and respiratory RSV iii. Cytology for CMV inclusions, fungi, and PCP iv. Direct fluorescence staining with antibodies against CMV, RSV, HSV, VZV, influenza virus, parainfluenza virus, adenovirus, and other organisms c. Other organisms/tests to also consider: i. Polymerase chain reaction for human metapneumovirus, rhinovirus, coronavirus, and HHV6 ii. Polymerase chain reaction for *Chlamydia*, *Mycoplasma*, and *Aspergillus* species iii. Serum galactomannan ELISA for *Aspergillus* d. Transbronchial biopsy if condition of the patient permits. III. Absence of cardiac dysfunction, acute renal failure, or iatrogenic fluid overload as etiology for pulmonary dysfunction

As centers adopt more advanced treatment planning techniques for TBI or total marrow irradiation (TMI), there is a greater need to understand the patient and treatment-related risks for IPS. In addition, standardized evaluation and reporting of IPS is crucial to compare outcomes between treatment techniques. Therefore, in this report, we critically evaluate the literature with the goal of characterizing the workup and definitions of pulmonary toxicity as well as levels of evidence in support of risk factors for IPS following TBI-based myeloablative stem cell transplant.

## Materials and Methods

A search was undertaken in PubMed, Embase, and Cochrane Library. Articles from 1960-2020 were searched using terms including “total body irradiation”, or “whole body radiation” and “radiation pneumonias” or “interstitial pneumonia” and “bone marrow transplantation” (**Supplemental Table 1**). Only English language reports of myeloablative transplant regimens were included. Studies in which the dose of TBI was not reported or intensity modulated techniques were used were omitted. Studies in which the incidence or risk factors for pneumonitis from TBI based regimens were not separately reported from those using chemotherapy alone were omitted.

Abstracted data included patient clinical characteristics such as age and disease; treatment-related characteristics including conditioning regimen, donor source, and graft *versus* host disease (GVHD) prophylaxis; TBI parameters including dose, dose rate, lung shielding, and beam arrangement; and outcomes including rates of acute GVHD.

We evaluated definitions of pulmonary complications in each publication. Any pulmonary complication was classified as pulmonary toxicity (PT). Pulmonary complications specifically reported as idiopathic were classified as IPS. Evidence reported to support these diagnoses including radiographic criteria, infectious workup, and change in pulmonary function tests was documented. Distinctions between acute and late toxicity, grading scales, and rates and mortality from acute PT and IPS were abstracted.

Evidence quality supporting risk factors for PT and IPS were categorized as: level Ia (evidence from meta-analyses of multiple randomized controlled trials), level Ib (evidence from ≥1 randomized controlled trial), level IIA (evidence from ≥1 controlled study without randomization), level IIb (evidence from ≥ other quasi experimental study), level III (evidence from non-experimental descriptive studies such as comparative studies, correlation studies, and case-control studies), and level IV (evidence from expert committee reports or opinions and/or clinical experience of respected authorities).

## Results

### Clinical Characteristics

**Figure 1:**
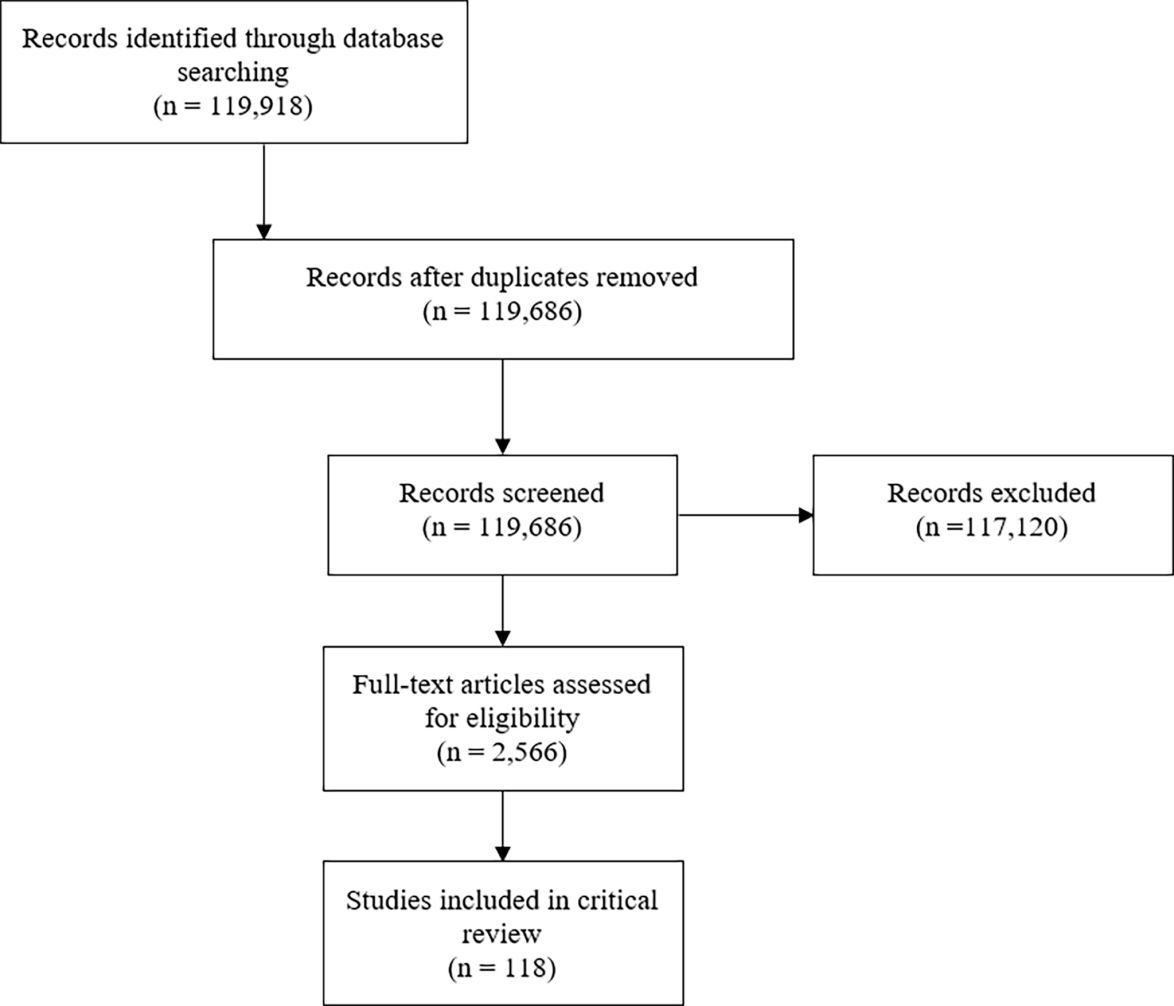
PRISMA diagram.

Of an initial 119,686 publications, 118 met inclusion criteria and were included for review (9, 12–127) (**Figure 1**). Studies were published between 1961-2020 and included patients from less than one year of age to 68 years of age (**Table 2**). Ten studies (8%) included patients with benign hematologic conditions and 17 included patients who received autologous stem cell transplants (16%). Most conditioning regimens were cyclophosphamide-based (88%) and most studies used methotrexate (MTX) for GVHD prophylaxis (57%). Rates of grade II-IV acute GVHD, when reported as such, ranged from 6-65%.

**Table 2:** Study characteristics.

Year (range)		1961-2020
Age (range, years)		<1-68
Conditions Included		
	Benign	10 (10%)
	Malignant	102 (99%)
Transplant Type		
	Autologous	17 (16%)
	Allogeneic	98 (95%)
Chemotherapeutic Backbone		
	Cyclophosphamide	91 (88%)
	Other/NR	12 (12%)
GVHD Prophylaxis		
	MTX	62 (60%)
	Other	14 (13%)
	NR	19 (18%)
	NA	5 (5%)
Rate of Acute Gr II-IV GVHD		7-65%
Definition of Pulmonary Toxicity		
	Interstitial pneumonitis	47 (45%)
Definition of Idiopathic Pulmonary Toxicity		
	Idiopathic interstitial pneumonitis	24 (23%)
Radiographic Criteria		
	Yes	46 (44%)
	No	57 (55%)
Infectious Workup		
	Yes	40 (38%)
	No	63 (61%)
Defined Acute *versus* Late		
	Yes	9 (9%)
	No	94 (91%)
Toxicity Grading Scale		
	None	84 (81%)
	CTCAE	7 (7%)
	RTOG	2 (2%)
	Individualized	10 (10%)

GVHD, graft versus host disease; MTX, methotrexate; NR, not reported; NA, not applicable; Gr, grade.

### Definitions

Separate definitions for PT and IPS were reported in 59 studies (50%). The most common definition for pulmonary toxicity was “interstitial pneumonitis” (45%) and for IPS was “idiopathic interstitial pneumonitis” (21%) (**Table 2**).

Forty-six (39%) of studies included or referenced a definition including radiographic criteria and 40 (34%) described an infectious workup including blood, sputum, BAL, or biopsy. Ten studies (8%) reported changes in pulmonary function tests.

A minority of studies differentiated between acute and late pulmonary toxicities (9%). Of these, 38% used a cutoff of 90 days, 31% a cutoff of 100 days, and 31% a cutoff greater than 6 months.

A grading scale for PT was provided in 20 studies (17%). Of these, 37% used Common Terminology for Adverse Events (CTCAE), 16% used Radiation Therapy Oncology Group (RTOG), and 47% used an individualized definition proposed by the study.

### Radiation Treatment Parameters

Of the studies evaluated, 59% reported the TBI source, 35% reported the beam arrangement, and 72% reported the dose rate. Of those that reported the source, 53% utilized Co-60 and of those that reported the beam arrangement 50% utilized anterior-posterior posterio-anterior (AP-PA) fields. Dose rate ranged from 1.2-30.0cGy/min.

Treatment fractionation was not described in 15% (**Table 3**). In 40% of studies patients were treated with a single fraction, most commonly to a total dose of 1000cGy (range 400-1754cGy). In 13% patients were treated with single daily fractions, most commonly to a total dose of 1200cGy (range 800-1575cGy). In 49% of studies patients were treated with twice daily fractions, most commonly to a total dose of 1200cGy (range 1020-1530cGy). In 4% of studies patients were treated three times per day, to a range of 1200-1610cGy.

**Table 3:** Dose and fractionation.

Fractionation	Dose (cGy)	N (%)
Fractionation not reported		19 (15)
	700-1440	1 (1)
	≥800	1 (1)
	≥900	1 (1)
	990-1600	1 (1)
	1000	1 (1)
	1000-1200	1 (1)
	1000-1440	1 (1)
	1000-1500	1 (1)
	1100-1400	1 (1)
	1125-1400	1 (1)
	1200	3 (3)
	1200-1500	1 (1)
	1200-1575	2 (2)
	1300-1375	1 (1)
Single treatment		47 (40)
	400-1505	1 (1)
	550	1 (1)
	550-900	1 (1)
	600	1 (1)
	700-850	1 (1)
	750	1 (1)
	750-900	1 (1)
	800	2 (2)
	800-1000	1 (1)
	950-1300	1 (1)
	900	1 (1)
	920	2 (2)
	1000	33 (28)
	1228-1754	1 (1)
Daily		15 (13)
	800	2 (2)
	900	1 (1)
	990	1 (1)
	1200	11 (9)
	1200-1500	1 (1)
	1400	1 (1)
	1575	3 (3)
Twice daily		58 (49)
	600-1200	1 (1)
	700-1100	1 (1)
	800-1200	1 (1)
	900-1200	1 (1)
	1000-1200	1 (1)
	1000-1320	1 (1)
	1000-1350	1 (1)
	1050-1400	1 (1)
	1100	2 (2)
	1100-1350	1 (1)
	1100-1320	1 (1)
	1200	34 (29)
	1200-1320	2 (2)
	1200-1360	2 (2)
	1200-1400	1 (1)
	1200-1700	1 (1)
	1320	7 (6)
	1320-1440	1 (1)
	1350	4 (3)
	1360	2 (2)
	1400	1 (1)
	1440	5 (4)
	1485	1 (1)
	1530	1 (1)
Three times daily		5 (4)
	1200-1610	1 (1)
	1320-1440	1 (1)
	1320	2 (2)
	1440	2 (2)

### Lung Shielding

Lung shielding techniques were not reported in 23% of studies evaluated (**Table 4**). In 26%, authors explicitly stated that no pulmonary shielding was used. In 42% of studies the lungs were shielded to a set mean dose, most commonly 800cGy (range 400cGy – prescription dose). Other studies reported pulmonary shielding by technique rather than dose limit, including use of 5-7 HVL blocks for a single treatment (3%), use of the patient’s arms (3%), or use of bolus, compensators, attenuators, or other unspecified custom blocks (8%).

**Table 4:** Lung shielding.

Technique or Dose	N (%)
Shielding not reported	27 (23)
No shielding	31 (26)
400cGy mean	1 (1)
500cGy mean	1 (1)
600cGy mean	2 (2)
730cGy mean	1 (1)
750cGy mean	2 (2)
800cGy mean	11 (9)
820cGy mean	1 (1)
850cGy mean	1 (1)
900cGy mean	8 (7)
1000cGy mean	10 (8)
1050cGy mean	4 (3)
1100cGy mean	2 (2)
1200cGy mean	4 (3)
Limit to prescription dose	2 (2)
10% shielding	1 (1)
40% shielding	1 (1)
50% shielding	1 (1)
Bolus/compensators/attenuators/custom blocks	10 (8)
5 HVL single treatment	1 (1)
6 HVL single treatment	2 (2)
7 HVL single treatment	1 (1)
Arms	3 (2)

### Pulmonary Toxicity

In studies where this was reported, PT occurred in 0-71% of patients treated with TBI, and IPS in 1-60%. Late PT occurred in 3-48% of patients treated with TBI and late IPS in 14-16%. Mortality from PT ranged from 0-61% and mortality from IPS ranged from 0-50%.

### Risk Factors for Pulmonary Toxicity

Fifty-three studies reported risk factors for PT (45%). Of these, the most frequently reported were GVHD (26%), older age (20%), increasing dose rate (13%), cytomegalovirus (8%), single fraction TBI (7%), and impaired pre-transplant pulmonary function tests (6%) (**Table 5**).

**Table 5:** Risk factors for pulmonary toxicity and IPS.

Risk Factors for Pulmonary Toxicity	N (%)
GVHD	14 (26)
Increasing age	9 (20)
Dose rate	7 (13)
CMV	4 (8)
Single fraction TBI	4 (8)
Impaired pre-transplant PFTs	3 (6)
Receipt of TBI	3 (6)
MTX	2 (4)
Performance status	2 (4)
Donor type	2 (4)
Lung dose/Lack of Shielding	2 (4)
Prior chemotherapy	1 (2)
>6 months from diagnosis to transplant	1 (2)
Prior radiation	1 (2)
T lymphocyte depletion	1 (2)
Infection	1 (2)
Non-CR at transplant	1 (2)
Co-60 based TBI	1 (2)
Diagnosis	1 (2)
Cyclosporine	1 (2)
Granulocyte infusion	1 (2)
Number of prior regimens	1 (2)
Graft failure	1 (2)
Year of BMT	1 (2)
AP-PA fields	1 (2)
Body weight	1 (2)
Prone position	1 (2)
Risk Factors for IPS	
Lung dose/Lack of Shielding	2 (10)
Dose rate	2 (10)
Receipt of TBI	2 (10)
Diagnosis	2 (10)
Myeloablative conditioning	1 (5)
CY dose	1 (5)
Anemia	1 (5)
CMV	1 (5)
Impaired pre-transplant PFTs	1 (5)
Parotitis	1 (5)
Single fraction TBI	1 (5)

GVHD, graft versus host disease; TBI, total body irradiation; MTX, methotrexate; CR, complete response; BMT, bone marrow transplant; AP-PA, anterio-posterior posterior-anterior; IPS, idiopathic pulmonary syndrome; CY, cyclophosphamide; CMV, cytomegalovirus.

### Risk Factors for IPS

Twenty-one studies reported risk factors for IPS. The most common risk factors were increased lung dose, increasing dose rate, receipt of a TBI containing regimen, and diagnosis (**Table 3**). Increased lung dose was associated with increased risk of toxicity in two studies (evidence level III). Increasing dose rate was associated with increased risk of pulmonary toxicity in two studies (evidence level III). Receipt of a TBI-containing regimen was associated with an increased risk of toxicity in two studies (evidence level III). Diagnosis was associated with increased risk of toxicity in two studies (evidence level III).

### Age Specific Considerations

Of the studies evaluated, 14 (12%) reported toxicity outcomes by patient age or stratified between adult and pediatric patients. Four of these studies found an increasing risk of PT with increasing age, one of which determined a cutoff of >20 years old (19, 20, 37, 43). Eighteen studies evaluated pediatric patients only, one of which demonstrated an increasing risk of IPS with dose rate >15cGy/min and one of which found an increased risk associated with chronic GVHD (69, 75).

## Discussion

In this study, we perform a critical review of existing literature defining and reporting the incidence of PT in patients who receive hematopoietic stem cell transplant with a myeloablative, TBI-based regimen. In our review, we find that rates of IPS may be as high as 60% with mortality as high as 50%. However, there are significant limitations in the existing literature defining and providing high-level evidence of risk factors for IPS.

### Defining IPS

Idiopathic pulmonary toxicity following TBI-based myeloablative transplant is thought to be due to direct injury to type II alveolar epithelial and endothelial cells from cells of lymphoid and myeloid origin as well as by inflammatory stimulators including TNF-α, lipo-polysaccharide, and reactive oxygen species (128–131). In mouse models of IPS, pulmonary toxicity due to host monocytes and donor T cells in the lungs occurs within the first 2 weeks of transplant (128). Increased cytotoxic T lymphocytes result in parenchymal damage and reduced compliance, total lung capacity, and increased wet and dry lung weights. In its advanced stages, IPS is characterized by cellular proliferation and matrix accumulation (132).

A standard clinical definition of IPS has been proposed (133). Criteria include evidence of widespread alveolar injury as evidenced by chest x-ray (CXR) or computed tomography (CT) scan, signs and symptoms of pneumonia, or abnormal pulmonary physiology, absence of active lower respiratory tract infection as diagnosed by BAL, transbronchial biopsy if feasible, and ideally a second confirmatory test to rule out infection. In this report, authors recommended against histopathologic definitions including “interstitial pneumonitis” citing concerns for accuracy. An expanded definition was proposed by the American Thoracic Society in which additional viral, fungal, and bacterial studies as well as evaluation of extra-pulmonary etiologies were recommended (10).

The studies evaluated for this critical review utilize multiple definitions of PT and IPS. The majority did not distinguish between idiopathic and non-idiopathic pulmonary complications. Of those that did, many relied on a definition of “interstitial pneumonitis” and less than half documented radiographic or other criteria supporting the diagnosis. Given that many studies had limited diagnostic workup, the true incidence of IPS secondary to an inflammatory-mediated process may not be accurately reported. In addition, varying definitions of IPS limit comparisons of the incidence between treatment regimens and evaluation of risk factors for IPS specifically as compared to other infectious pulmonary toxicity.

The working group publications do not make recommendations regarding definitions of acute and late toxicity, which is also reflected in the limited reporting and lack of consensus seen in the studies evaluated. In general, idiopathic pulmonary toxicity is thought to occur early after transplant, historically reported between six to seven weeks, although more recently as early as 19 days (133–135). In their studies, Kim et al. and Gao et al. restricted pulmonary events to those occurring within 90-100 days of transplant based on previous data demonstrating a maximal risk for IPS within the first 100 days of transplantation (77, 110). However, Nagasawa et al. evaluated pulmonary toxicities occurring 3 months or more after HSCT (hematopoietic stem cell transplant) (75). They found a 16% (4/25) incidence of late non-infectious pulmonary toxicities for patients receiving a TBI-based myeloablative regimen, suggesting that late pulmonary complications may occur frequently.

In newer studies using intensity modulated techniques, pulmonary toxicity has been preliminarily evaluated. However, definitions in these publications are also variable. In their study, Shinde et al. rigorously defined radiation pneumonitis as greater than or equal to grade 3 pneumonitis not attributable to infection, graft *versus* host disease, or disease progression as assessed by standard institutional post-HCT protocols including bronchoscopy (136). Others have reported only rates of any pulmonary toxicity without specifying workup or etiology (137, 138).

### Recommendations for Definition

We recommend routine adoption of the diagnostic workup and definition of IPS proposed by the American Thoracic Society. Based on the available literature and previously used definitions we suggest a cutoff of early toxicity within 90 days of transplant and encourage continued patient follow-up for late treatment-related toxicity.

### Grading IPS

Toxicity grading scales were not commented on in the working group publications and infrequently utilized in the papers studied for this review. Seven studies relied on CTCAE definitions and two utilized Radiation Therapy Oncology Group (RTOG) definitions (47, 55, 73, 81–83). Five defined toxicity with an individualized scale, one of which utilized the extent of imaging changes on CXR (41, 44, 52, 97). In studies without a grading scale, pulmonary toxicity is most often reported as present and resolved or a cause of mortality. Notably, no patient reported outcomes (PRO) were utilized in these studies, although PRO surveillance using the PRO-CTCAE has been shown to be feasible in the transplant setting (139). Studies using 3D treatment planning have relied on multiple grading scales including CTCAE and Bearman Toxicity Scale for bone marrow transplantation (136–138, 140).

### Recommendations for Grading

There is little data to suggest benefit to one grading scale over another. However, given the availability of both provider and patient-reported outcomes, authors encourage consideration of the CTCAE for future toxicity reporting.

### Risk Factors for IPS

The studies reporting risk factors for IPS were, in general, lower levels of evidence. Limitations of these publications included heterogeneous diagnoses for which patients may have received previous chemotherapy and/or radiation, wide age ranges, multiple conditioning regimens, approaches to GVHD prophylaxis, and a variety of donor sources which all impact the risk of complications and could not be controlled for in evaluation of risk factors for PT or IPS.

The details of TBI and cytotoxic chemotherapy were not uniformly reported and limit the ability to evaluate safety and efficacy between varying chemotherapy regimens, doses, dose rates, beam arrangements, and shielding techniques. Cytotoxic chemotherapy may result in pneumonitis without the addition of radiation, and few studies provided the regimen and doses of chemotherapy delivered in conjunction with TBI (141). In addition, all studies are limited by the accuracy of true lung dose assessment. The majority of TBI patients are treated with either right and left lateral or AP-PA fields without 3D treatment planning. Details of the techniques have been previously described (51, 142). It is difficult to assess the limiting lung dose, due particularly to inaccuracies in the largely inhomogeneous dose distribution within the lung resulting from the single-point dose calculation model generally used (143, 144). The CT based treatment planning simulation showed a highly inhomogeneous dose distribution from TBI delivery to different organs. The greatest variation in radiation dose in the lung was as much as 32% above that prescribed (145). Therefore, the exact correlation of the single point dose and lung pneumonitis from the reported studies will have to be carefully considered.

In spite of these limitations, some risk features were seen in multiple studies which may inform radiation planning parameters. Dose rate was found to be significantly associated with development of IPS in several non-randomized studies (**Table 6**). Abugideiri et al. evaluated 129 pediatric patients who underwent TBI-based myeloablative conditioning at dose rates from 2.6cGy/min to 20.9cGy/min and found a statistically significant association of dose rate with IPS on multivariate analysis (*p*=0.002) (69). Gao et al. evaluated 202 patients with acute leukemia at a dose rate from 8.7cGy/min to 19.2cGy/min. Patients treated with high dose rates, defined as >15cGy/min, had a 29% incidence of IPS as compared to 10% in those patients treated with lower dose rates (*p*<0.01). In a retrospective study of 92 patients with hematolymphoid malignancies treated with 900-1200cGy fractionated TBI, a trend towards decreased toxicity was seen in those treated at a lower dose rate, defined as <6cGy/min (*p*=0.07) (110). In their analysis, Barrett et al. found that dose rate had an effect on PT only at total lung doses of >900cGy without an effect at lower total doses (80).

**Table 6:** Summary of key literature reports on the effect of dose rate and lung dose.

First author, publication year	*N*	Prescribed dose (Gy)/fx	Lung dose (Gy)	Dose rate (cGy/min)	Findings
Barrett, 1982 (80)	402*	7.5-10.5/1	1-12	2.5-46	Dose rate associated with incidence of PT only for lung dose ≥9 Gy.
Bortin, 1982 (95)	176*	≥8	NR	2.3-30	Dose rate ≤5.7 cGy/min associated with lower risk of PT 30% *vs*. 6%)
Weiner, 1986 (19)	932^	10/1 or 12/5-6	5.6-12.8	2-108	Dose rate significantly correlated with risk of PT only in those receiving MTX after transplantation.
Ozsahin, 1996 (43)	186^	10/1 or 12/6 BID	8 or 9	2.6-16.9	PT incidence was significantly higher in the high dose rate patients – 56% (> 9cGy/min) *vs*. 20% (≤4.8 cGy/min).
Corvo, 1999 (89)	93^	12/6 BID	10.8 or 12	2.5-15	PT incidence was correlated with higher dose rate – 33% (>6 cGy/min) *vs*. 12% (<6 cGy/min).
Carruthers, 2004 (53)	84^	12/6	NR	7.5 and 15	A higher dose rate associated with a higher risk of PT - 43% (15 cGy/min) *vs*. 13% (7.5 cGy/min).
Abugideiri, 2016 (69)	129^±^	10.5-14 (1.5-2 Gy/fx)	≤ 10	5.6-20.9	TBI dose rate ≥15 cGy/min significantly increased incidence of PT [HR 4.85] and IPS [HR 4.94].
Kim, 2018 (17)	92^	9-12/3-4 daily	5-10% attenuation	4.2-17.3	Reducing the dose rate decreased the risk of PT - 74.1% (≥6 cGy/min) *vs*. 43.5% (<6 cGy/min).
Gao, 2019 (77)	202^	13.2/8 BID	None	8.6-19.2	IPS in 29% (>15cGy/min) *vs* 10% (≤15cGy/min).
Petersen, 1992 (87)	36^	12 or 16/6 BID, 17/7 BID	Prescription	8	PT or IPS in 50% of patients receiving 17Gy as compared to 15% after 16Gy.
Sampath, 2005 (91)	1090*	Up to 15.6	Up to 15.6	3-41	Lung dose was associated with PT in patients receiving 1 fx/day – 2.3% if ≤6 Gy to lungs.
Soule, 2007 (57)	181^≠^	12 or 13.6 (1.5-1.7 Gy/fx bid)	6 to >13.6	12	Lung dose reduction should be employed primarily to decrease mortality from PT in high-risk patients.
Weschler, 1990 (30)	43^	6/4 BID (TLI) or 12/6 BID (TBI)	6 or 12	15-18	IPS occurred in 26% without lung shielding as compared to 0% with partial lung shielding.

bid, twice a day; fx, fraction; HR, hazard ratio; IP, interstitial pneumonitis; IPS, idiopathic pneumonia syndrome; MTX, methotrexate; NR, not reported; PT, pulmonary toxicity; TBI, total body irradiation; TLI, total lymphoid irradiation; *ages not specified; ^adults and children; ^±^children; ^≠^adults and adolescents.

Higher lung dose has also been found to be associated with IPS at a range of dose rates (**Table 7**). Weshler et al. evaluated 44 patients with malignant disorders treated with TBI containing myeloablative transplants (30). Their first 23 patients were treated to 1200cGy with fractionated TBI without lung shielding at a dose rate of 15-18cGy/min and found a 26% risk of IPS. The remainder were treated with a 50% transmission lung block at a dose rate of 15cGy/min with no IPS. Petersen et al. performed a phase I dose escalation trial utilizing TBI given at a rate of 8cGy/min in 200cGy fractions twice daily from 1200-1700cGy without lung shielding. They found 50% risk of PT at a dose of 1700cGy as compared to 15% after 1600cGy (87). Sampath et al. similarly evaluated 20 articles to develop a multivariate logistic regression to determine dosimetric and chemotherapeutic factors influencing the incidence of IPS (91). In their analysis, a conditioning regimen of 1200cGy fractionated TBI resulted in an incidence of IPS of 11% as compared to 2.3% with 50% lung shielding (*p*<0.05) without any effect from dose rate.

**Table 7:** Summary of key literature reports on the effect of lung dose.

First author, publication year	*N*	Prescribed dose (Gy)/fx	Lung dose (Gy)	Dose rate (cGy/min)	Dose rate finding
Petersen, 1992 (87)	36^	12 or 16/6 BID, 17/7 BID	Prescription	8	PT or IPS in 50% of patients receiving 17Gy as compared to 15% after 16Gy.
Sampath, 2005 (91)	1090*	Up to 15.6	Up to 15.6	3-41	Lung dose was associated with PT in patients receiving 1 fx/day – 2.3% if ≤6 Gy to lungs.
Soule, 2007 (57)	181^≠^	12 or 13.6 (1.5-1.7 Gy/fx bid)	6 to >13.6	12	Lung dose reduction should be employed primarily to decrease mortality from PT in high-risk patients.
Weschler, 1990 (30)	43^	6/4 BID (TLI) or 12/6 BID (TBI)	6 or 12	15-18	IPS occurred in 26% without lung shielding as compared to 0% with partial lung shielding.

bid, twice a day; fx, fraction; IP, interstitial pneumonitis; IPS, idiopathic pneumonia syndrome; NR, not reported; PT, pulmonary toxicity; TLI: total lymphoid irradiation; *ages not specified; ^ adults and children ^≠^adults and adolescents.

New advancements using CT guided intensity modulated TBI or TMI or TMLI allow 3D lung dose distribution to be calculated with high precision (146, 147). Reported rates of pneumonitis are low in spite of dose rates up to 200cGy/min (136, 148). Clinically, a mean lung dose of 800cGy or less has still been associated with decreased risk, suggesting need for further study using 3D planning to understand the relationship between dose rate, mean lung dose, and IPS (136).

There are limited data regarding differences in pulmonary risk and modifications to treatment planning that should be made based on patient age. In many of the studies evaluated, increasing age was found to be a risk factor for pulmonary toxicity. This may be due to worse pre-transplant pulmonary function, which has been found to be a risk factor for IPS (79). However, pediatric patients remain at risk of pulmonary toxicity. In a study of only pediatric patients, increasing dose rate was found to be a risk factor for IPS (69). Similarly, increasing mean lung dose, while not correlated with risk of IPS in any study of only pediatric patients, was correlated with reduced survival (100). TBI using intensity modulated techniques has been reported in children and young adults without any early evidence of increased risks of toxicity (149). However, longer follow up, larger patient numbers, and more comprehensive dosimetric evaluation is needed in order to obtain pediatric-specific planning parameters.

### Recommendations for Dose Rate and Shielding

Risk factors for true IPS remain poorly defined given limitations in definitions, workup, and reporting of TBI parameters. A dose rate of ≤15cGy/min and a mean lung dose ≤600cGy using traditional planning techniques is supported by the literature.

### Future Directions

As more centers adopt 3D image guided intensity modulated treatment planning for TBI to reduce the lung dose, quantitative knowledge of how dose distribution and lung volume coverage may be correlated with IPS should emerge. Consequently, standard methods of evaluating and reporting pulmonary toxicity will become even more critical. Efforts to adopt the definition and workup for IPS proposed by the NIH and American Thoracic Society are needed in addition to further studies of differences in risk factors and clinical outcomes between pediatric and adult patients and a greater understanding of the contribution of specific chemotherapy regimens to the overall risk. More comprehensive and reliable reporting of TBI dosimetric parameters will provide greater understanding of the range of treatment and planning techniques and their relationship to IPS.

## Author Contributions

JV, SH, and NE contributed to study design, data analysis, manuscript writing, and manuscript review. C-HH contributed to manuscript review and data presentation. KD, PR, JK, and LC contributed to manuscript review. All authors contributed to the article and approved the submitted version.

## Funding

The work was partially supported by the National Institutes of Health under R01CA154491 (SH).

## Conflict of Interest

The authors declare that the research was conducted in the absence of any commercial or financial relationships that could be construed as a potential conflict of interest.

## Publisher’s Note

All claims expressed in this article are solely those of the authors and do not necessarily represent those of their affiliated organizations, or those of the publisher, the editors and the reviewers. Any product that may be evaluated in this article, or claim that may be made by its manufacturer, is not guaranteed or endorsed by the publisher.
