# Supplementary material for: Pulmonary Toxicity After Total Body Irradiation – Critical Review of the Literature and Recommendations for Toxicity Reporting
Source: Front Oncol. 2021 Aug 26;11:708906. doi: 10.3389/fonc.2021.708906 (PMC8428368; doi:10.3389/fonc.2021.708906)
Supplement: Supplementary file 1 [file DataSheet_1.pdf]

Supplemental Table 1: Search Terms

| | | |
|---|---|---|
| Pneumonitides, radiation | Whole body irradiation | Stem cell transplantation, hematopoietic |
| Radiation pneumonitides | Irradiation, whole body | Transplantation, hematopoietic stem cell |
| Pneumonia, radiation | Irradiations, whole body | Bone marrow transplantation |
| Radiation pneumonia | Whole body irradiations | Transplantation, bone marrow |
| Pneumonias, radiation | Irradiation, total body | Grafting, bone marrow |
| Radiation pneumonias | Total body irradiation | Bone marrow grafting |
| Pneumonitis, radiation | Irradiations, total body | Bone marrow cell transplantation |
| Radiation pneumonitis | Total body irradiations | Transplantation, bone marrow cell |
| Fibrosis, radiation | Radiation, whole body | |
| Radiation fibrosis | Whole body radiation | |
| | Radiations, whole body | |
| | Whole body radiations | |
